# Ischemic Stroke and Epilepsy in a Patient with Tourette´s Syndrome: Association with the Antiphospholipid Syndrome and Good Response to Levetiracetam

**DOI:** 10.2174/1874205X00802010032

**Published:** 2008-06-12

**Authors:** M Seijo-Martínez, J.A Mosquera-Martínez, S Romero-Yuste, J Cruz-Martinez

**Affiliations:** 1 Neurology Service, Hospital do Salnés, Spain; 2 Rheumatology Service, Complexo Hospitalario de Pontevedra, Spain

**Keywords:** Tourette syndrome, antiphospholipid syndrome, antiphospholipid antibodies, levetiracetam

## Abstract

The role played by different humoral factors, including antiphospholipid antibodies, in the pathogenesis of Tourette syndrome (TS) is still presently unclear. We present a patient with chronic and severe TS who, at the age of 16 years, presented an ischemic stroke in the left posterior cerebral artery and/or postero-inferior temporal branch of the left medial cerebral artery. A complete study was negative with the exception of a positive lupus anticoagulant. The stroke was related with the primary antiphospholipid syndrome (APS). The stroke manifested visual abnormalities and thereafter by secondary generalized complex partial seizures. The epileptic syndrome was initially difficult to control but responded dramatically to levetiracetam. With this therapy, the manifestations of TS, especially the tics, improved. We conclude that some TS cases may present APS. In addition, levetiracetam may be useful in the management of TS. Further investigations should pursue both these facts.

## INTRODUCTION

The pathogenesis of the Tourette syndrome (TS), the main component of the primary tic-syndrome [1], is presently unclear. Both genetic and non-genetic factors are important considerations [2-8]. The antiphospholipid antibodies (aPLAs) are a group of heterogeneous antibodies which bind with negatively charged phospholipids. When present in association with vascular thrombosis or recurrent fetal loss, the diagnostic criteria of the antiphospholipid syndrome (APS) are fulfilled [6]. The neurological complications of APS are varied and include movement disorders and chorea. However, the mechanism underlying non-thrombotic movement disorders in APS remains presently unknown. A direct immune interaction of aPLAs with diverse brain structures has been hypothesized. Presently, there is conflicting evidence of an association of aPLA with TS [7-8].

Herein, we present a young patient with severe TS who presented an ischemic stroke in association with APS and secondarily presented epileptic syndrome. We reappraise the hypothesis that aPLAs may play a role in TS. In addition, we highlight the fact that levetiracetam therapy had a significant beneficial effect in the control of the tics and the epileptic syndrome.

## CASE REPORT

**Fig. (1):**
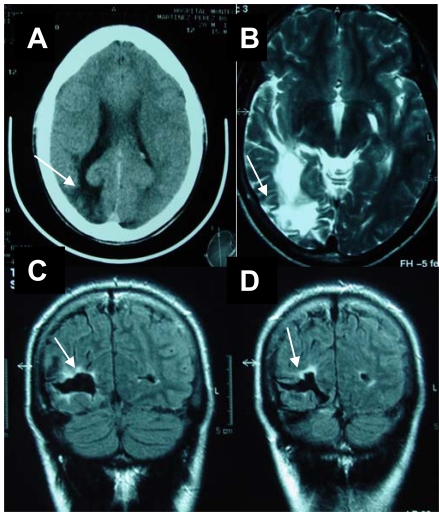
**a.** Brain CT. Hypodense right occipital lobe lesion (arrow) with dilatation of the ventricular horn and widened cortical sulci (atrophy). **b.** Brain MRI (FLAIR). Axial section. Hyperintense white matter signal in the occipital lobe (arrow), ventricular dilatation and wid-ened sulci. **c-d.** Brain MRI (T2 weighted). Coronal section. Hypointense signal in the right occipital lobe with a peripheral hyperintensity (arrow) suggestive of a chronic ischemic infarct.

The patient is presently a 25 year-old male with a family history for a transient tic-disorder present in the infancies of both his father and paternal grandfather. He was born after an uneventful pregnancy and birth. From the age of 8 years he presented a progressive and severe complex motor and vocal tic disorder including echolalia, palilalia and coprolalia in association with obsessive-compulsive behavior. He was diagnosed of TS. The severity of the syndrome required multiple evaluations over the years in various neurological centers and by the psychiatry department. He was treated with multiple combination therapies with neuroleptics, anticholinergics and tricyclics but the clinical response was basically poor. The tic disorder had a severe and negative impact in his family, social relations and on his academic results. In this period, an extensive and complete study including neuroimaging studies with CT and MRI were normal. At the age of 16 years, he presented sudden-onset blurring in his left hemifield followed by a tonic-clonic seizure of minutes´s duration. No motor, sensory or language abnormalities were present. The brain CT and MRI showed the presence of a right parietal-occipital ischemic stroke (Fig. **1a**,**b**,**c**,**d**). An angio-MRI of the brain and neck vessels was normal. An electrocardiogram and trans- esophageal echocardiogram were normal. Thyroid function and coagulation studies were normal. In the serum, titers of nuclear antibodies (ANAs), performed by indirect inmunofluoresce using rat liver and mouse kidney were positive with 1/640 titer and the anti-DNA, antihistone, anti-SSA, anti-SSB and anti-RNP antibodies were negative; C3 and C4 serum levels were normal. The syphilis serology and anticardiolipin antibodies were negative. The lupus anticoagulant was positive (dRVVT, Instrumentations Laboratory) on two tests performed in an interval of 12 months.

The left-sided visual abnormalities improved over a few weeks. However, the seizure disorder persisted. The seizures were fairly stereotyped and consisted in paroxistic onset of an oppressive epigastric sensation, tachycardia, diaphoresis and visual alterations. These visual phenomena were rich and varied in type: metamorphopsia, positive phenomena of complex nature in the left hemifield, and a vague sensation described as “far vision”. Some of these episodes were followed by a head turning to the left and impaired consciousness and confusion of variable but, generally brief, duration. He was diagnosed of secondary generalized complex partial (autonomic, visual and versive) epilepsy and the patient was managed with antiepileptic drugs (AED) in various combinations (valproate, carbamacepine, phenobarbital and phenytoin). These agents were mainly effective on the generalized convulsive fits, but the other phenomena persisted, were frequent (2-4 per week) and motivated many consultations in the emergency room.

At the age of 21 years levetiracetam 1000mg bid was initiated as add-on therapy to phenytoin 300 mg/day and the seizure disorder came under complete and total control. Simultaneously, with the initiation of levetiracetam, the tic-disorder (both the motor and vocal tics) improved in parallel with the control of the seizure disorder. The patient maintains treatment with platelet inhibitors and levetiracetam; the dose of phenytoin is being progressively lowered (presently at 100 mg/day). He is seizure-free since the initiation of levetiracetam therapy.

The patient still presents occasional vocal and motor tics but is able to lead a satisfactory personal and social life. In addition, he has earned an employment as a public servant.

## DISCUSSION

This patient with severe TS presented an ischemic stroke in the posterior cerebral artery and/or postero-inferior temporal branch of the right medial cerebral artery. A stroke work-up was negative with the exception of a positive lupus anticoagulant repeated on various occasions. Systemic diseases were excluded and the patient satisfied the diagnostic criteria for primary APS [6]. The stroke manifested visual abnormalities and secondary generalized complex partial seizures. Upon reaching a good control of the seizures the manifestations of the TS improved.

The pathogenesis and pathophysiology of TS are still presently unknown. Abnormalities in both the dopaminergic and, to a lesser extent, serotoninergic neurotransmission within the basal ganglia and the cortico-ganglionic-thalamo-cortical circuitry have been suggested [2].

Both genetic and non-genetic factors may play a role in the pathogenesis of TS. The presence of certain cytogenetic abnormalities, including chromosomal translocations and inversions, and epigenetic risk factors suggest that certain genetic or chromosomal irregularities may be common to some cases of TS [2]. There is also evidence for an immunological basis in TS. Anti-basal ganglia antibodies appear to play a role in certain TS-spectrum disorders including the pediatric autoimmune neuropsychiatric disorders associated with streptococcal infection (PANDAS). These antibodies have been found in 65% of patients with atypical movement disorders [3]. Anti-basal ganglia antibodies are often found in children with Sydenham´s chorea. In this disease, antibodies seem to attack the basal ganglia by a mechanism of molecular mimicry triggered by a previous streptococcal infection [8]. In TS there are higher concentrations of anti-putaminal antibodies compared to normal controls [5] and immunomodulatory treatment is effective in some patients. There are therapies that may act on a putative immune-mediated attack directed against the nigrostriatal or prefrontal dopaminergic pathways [3,9].

Some studies have approached the possible association between aPLAs and TS. Antiphospholipid antibodies have been identified in children with TS and have led to the speculation that these antibodies might play a pathophysiological and possibly pathogenic role in this condition [7]. If confirmed, the presence of aPLAs has diagnostic and therapeutic implications in TS. However, aPLAs in TS may represent an epiphenomenon rather than a pathophysiological mechanism [8]. Unfortunately, the number studies on this aspect are scarce and have not included patients with APS.

To our knowledge, this is the first report of a TS patient presenting a stroke in association with APS. Acknowledging a possible chance association, in some TS cases immune phenomena may be involved in the movement disorders. The presence of a particular genetic load or predisposition may increase susceptibility in developing specific antibodies and thrombotic phenomena.

Also of interest in this case is the favorable response of both the epileptic syndrome and the tic-disorder to levetiracetam. It is possible that the seizure disorder may have aggravated the frequency and severity of the tics but we do not exclude a direct effect of levetiracetam on the favorable outcome of the tic-disorder [10].

In conclusion, this report indicates that aPLAs may be present in some patients with TS. We believe that the mechanisms that trigger the generation of these antibodies leading to vascular events and possibly movement disorders should be further investigated.
